# 

**Published:** 1873-01

**Authors:** 


### INDEX.

A PAGE

Abortionists, To ........ 440
Abuse of Medical Charities ........ 632
Adolphus, Philip, M.D., Present Status of the Pessary in Gynecological Practice 18
Case of Congenital Variola in Fœtus .. 278
Air, Disinfection of—A. Ernest Sansom, M.D ........ 279
Alcohol, Physiological Relations of—W. Hay, M.D ........ 386
Allen Nathan, Law of Population ........ 332
Amalgam Plugs, Corrosive Sublimate generated by—J. Payne, D.D.S ........ 397
Amalgam Plugs—W. Henry King, M.D. 523
Amalgam Plugs—Burke Pillsbury, M.D. 596
American Medical Association, Report of ........ 251, 364
Amnios, Dropsy of—J. W. B. Reynolds, M.D ........ 54
Anatomical Relations of Pulmonary Phthisis to Tubercle—Discussion in Pathological Society, London—Z. Collins McElroy, M.D ........ 723
April Fooled—L. B. Brown, M.D ........ 527
Asiatic Cholera—H. A. Buck, M.D ........ 461

B

Barking of the Nose ........ 668
Bartlett, John, M.D., The Cervix Uteri, Before, During, and After Labor ........ 577
Bates, X. T., Elixir Iodo-Bromide of Calcium Compound in Scrofulous Induration of the Nose ........ 396
Bed "Sores," Clinical Lecture on—Sir James Paget ........ 424
Bigelow, H. R., M.D., On Paresis ........ 403
Hamlet's Insanity ........ 513
Premonitory Symptoms of Insanity ........ 717
Bill to Promote Science of Medicine and Surgery in Illinois ........ 126, 758
Blake, C. J., Effect of the Galvanic Current upon the Auditory Nerve ........ 494
Blood, Dry-Powdered—Dr. De Pascale .. 435
Book Notices. See Editors' Book Table.
Boone, H. W., M.D., Physical Education and Training of Children ........ 38
Brain, Diseases of—C. E. Brown-Sequard 468
Brain, Methods of Examining the—Jas. J. Putnam, M.D ........ 683
Breast, Cancer of Female ........ 597
Bromine in Croup ........ 675
Bromo-Chloralum in Scirrhous Affections of the Stomach ........ 398
Brown, S. B., M.D., April Fooled ........ 527
Buck, H. A., M.D., Asiatic Cholera ........ 461

C

Calabar Bean in Traumatic Tetanus—J. A. Duncan, M.D ........ 686
Cancer of the Female Breast ........ 597
Capital Punishment ........ 636
Carditis, Rheumatic, Case of—H. A Johnson, M.D ........ 500
Central Illinois Medical Society ........ 127
Certificates of Family Physicians—W. C. Wey, M.D ........ 562
Cervix Uteri, Before, During, and After Labor—John Bartlett, M.D ........ 577
Charities, Abuse of Medical ........ 632

PAGE

Chicago Society of Physicians and Surgeons ........ 146, 385, 590, 742
Chicago Medical Journal, Historical Memoranda of ........ 753
Children, Physical Education and Training of—H. W. Boone, M.D ........ 38
Children, Clinical Examination of—D. N. Kinsman, M.D ........ 286
Children, Medical Care and Nursing of—Jerome Walker, M.D ........ 290
Cholera—Walter Hay, M.D ........ 442
Cholera Infantum—Alex. S. von Mansfelde, M.D ........ 453
Cholera, Asiatic—H. A. Buck, M.D ........ 461
Cholera ........ 573
Cholera—Latest Discussions in Germany on the subject ........ 660
Chronic Inflammation of the Stomach ... 585
Civil Malpractice — M. A. McClellan, M.D ........ 65, 129, 193
Clavicle, Fracture of ........ 703
Coffee, Infusion of Green Coffee in Gout 688
Compressed Air—J. H. Etheridge, M.D. 166
Concours, The, Rush Medical College .. 64
Cook County Hospital ........ 571
Cord, Prolapsus of the—J. E. O'Brien, M.D ........ 656
Criminals, Are Lunatics? ........ 700
Crothers, T. D., M.D., Sanitary Statistics of England ........ 433
Croup, Bromine in ........ 675

D

Dawson, B. F., M.D., Whooping Cough and Quinine ........ 224
De Profundis Clamavi—"Out of the Sewers" ........ 383
De Witt Co. Medical Society ........ 448
Diarrhœa, Infantile—Ira E. Oatman, M.D 336
Diet, Night ........ 119
Diphtheritic Ophthalmia — F. C. Hotz, M.D ........ 32
Discussion on Anatomical Relations of Pulmonary Phthisis to Tubercle, in Pathological Society, London—Z. Collins McElroy, M.D ........ 723
Disinfection of Air — A. Ernest Sansom, M.D ........ 279
Dropsy of the Amnios—J. W. B. Reynolds, M.D ........ 54

E

Ear, Clinical Lectures on Diseases of—E. L. Holmes, M.D ........ 1, 321
EDITORS' BOOK TABLE:
Adams, Club Foot ........ 244
Allingham, Fistula and other Diseases of the Rectum ........ 246
Anderson, On Diseases of the Skin. 59, 507
Attfield, Chemistry ........ 506
Atthill, Clinical Lectures ........ 58
Barnes, Gen. Jos. K., Medical and Surgical History of the War of the Rebellion ........ 186
Bell's Anatomy and Philosophy of Expression ........ 307
Bennett's Text Book of Physiology ... 306

Editors' Book Table— PAGE
Bigelow, The Physiology and Psychology of the Brain ... 628
Bloxam's Chemistry ... 693
Both, On Small-Pox ... 60
Braithwaite, Retrospect ... 123
Bryant, Surgery ... 121
Byford, Theory and Practice of Obstetrics ... 247
Davis, N. S., Clinical Lectures ... 308
Dental Caries and Causes ... 185
Dieulafoy, Treatise on the Pneumatic Aspiration of Morbid Fluids ... 696
Ecker, The Cerebral Convolutions of Man ... 630
Elmer, Physicians' Hand Book ... 60
Erichsen, Science and Art of Surgery. 186
Flint's Practice of Medicine ... 381
Fox, Skin Diseases ... 630
Frey, Microscope and Microscopic Technology ... 60, 243
Garretson, Oral Surgery ... 59
Gouley, Urinary Organs ... 307
Greave, Questions in Surgery ... 58
Guersant, Surgical Diseases of Children ... 123
Hamilton, Clinical Electro-Therapeutics ... 749
Hammond, Nervous Diseases ... 59
Hammond, Insanity and its Relations to Crime ... 697
Hazard, Santo Domingo ... 247
Helmholtz, Mechanism of the Ossicles of the Ear and Membrana Tympani ... 381, 507
Hewitt, Diseases of Women ... 59
Hodge, On Fœticide ... 123
Hoffman, Chemical Analysis ... 244
Holbrook, Parturition without Pain ... 122
Keen's Diagrams ... 58
Kirkes' Hand Book of Physiology ... 749
Klein's Hand Book for the Physiological Laboratory ... 380
Lidell, On Apoplexy, etc ... 246
McClelland's Civil Malpractice ... 308
Maudsley, Body and Mind ... 629
Moorman, Mineral Springs of North America ... 631
Norris, Contributions to Practical Surgery ... 699
Parkes' Manual of Practical Hygiene. 692
Piffard, a Guide to Urinary Analysis ... 505
Ray, Contributions to Mental Pathology ... 244
Report of the Columbia Hospital ... 627
Roberts, Urinary Organs ... 59
Roosa, Diseases of the Ear ... 751
Seguin, Manual of Thermometry ... 505
Sheppard, Lectures on Madness ... 697
Stellwag, Diseases of the Eye ... 750
Stille, Epidemic or Malignant Cholera. 631
Sturgis, Clinical Medicine ... 506
Swayne, Obstetric Aphorisms ... 123
Sweringen, Pharmaceutical Lexicon ... 699
Taylor's Medical Jurisprudence. 689, 750
Thompson, Diseases of the Prostate ... 748
Tibbitt's Hand Book of Medical Electricity ... 38
Transactions of Am. Med. Association 752
Trousseau's Clinical Medicine ... 691
Tuke, Influence of the Mind upon the Body ... 245
Tuke, Illustrations of the Influence of the Mind upon the Body ... 437
U. S. Pharmacopœia, 1873 ... 121
Wells, Diseases of the Eye ... 752

Editors' Book Table— PAGE
Walton, Mineral Springs of the U. S. and Canada ... 631
Wells, Diseases of the Ovaries ... 122
Wohler, Organic Chemistry ... 122
Woodworth, Jno. M., M.D. Annual Report ... 185
Electro-Thermal Bath, Therapeutics of —Justin Hayes, M.D ... 641
Elixir Iodo-Bromide of Calcium Compound in Scrofulous Induration of the Nose—X. T. Bates, M.D ... 396
England, Sanitary Statistics of—T. D. Crothers, M.D ... 433
Epidemic Cerebro-Spinal Meningitis. 269, 345
Erysipelas, Iodide of Potassium in—A. H. Hiatt, M.D ... 593
Etheridge, J. H., M.D., On Compressed Air ... 166
Translations from Foreign Journals ... 668
Exophthalmic Goitre, The Ophthalmoscopic Appearances in Cases of—R. A. Vance, M.D ... 449

F

Facial Paralysis, Case of—G. M. Smith, M.D ... 560
Family Physicians, Certificates of — W. C. Wey, M.D ... 562
Faradaic Electricity — Plym. S. Hayes, M.D ... 649
Feeble-Minded Children, Illinois Institution for ... 576
Fees versus Salaries ... 187
Females, Retreat for Insane ... 61
Female Breast, Cancer of ... 597
Femur, Shortening and Bending in Fractures of—J. E. Nichols, M.D ... 275
Flint, Austin, M.D., On Acute Articular Rheumatism ... 357
Food, Protracted Abstinence from—A. Ford, M.D ... 460
Ford, A., M.D., Protracted Abstinence from Food ... 460
Fracture of the Clavicle treated ... 703
Freer, J. W., M.D., Septicæmia ... 10

G

Galvanic Current upon the Auditory Nerve—C. J. Blake ... 494
Gelseminum as an Antiperiodic — Wm. W. Murray, M.D ... 355
Glass, Injury from—George W. Kittell, M.D ... 324
Gout, Cold Infusion of Green Coffee in ... 688
Grafting, Skin ... 63
Gynecological Practice, Present Status of Pessary in—Philip Adolphus, M.D 18

H

Hamlet's Insanity—H. R. Bigelow, M.D. 513
Hand, H. C., M.D., Phthisis as related to Syphilis and Scrofula ... 411
Hay, Walter, M.D., On Neuralgia ... 4, 97
Appointed Professor ... 64
Physiological Relations of Alcohol ... 386
On Cholera ... 442
To Abortionists ... 440
On Demise of Brown-Sequard's Journal 757
Hayes, Justin, M.D., Therapeutics of the Electro-Thermal Bath ... 641
Hayes, Plym. S., M.D., Report of Transactions of the Chicago Society of Physicians and Surgeons ... 385, 590, 742
Faradaic Electricity ... 649
Head of the Firm gone East ... 508

PAGE

Hemorrhage, Umbilical, Death from—Norman Bridge, M.D. .......... 595
Hernia, Femoral, Strangulated—J. Suydam Knox, M.D. .......... 458
Historical Memoranda of this Journal .. 753
Holmes, E. L., M.D., On the Ear.....1, 321
  Rare Abnormal Condition of the Iris .. 330
Homœopathy, What is? .......... 318
Homœopathy in the Michigan University 384
Horse Influenza .......... 320
Hotz, F. C., M.D., On Diphtheritic Ophthalmia .......... 16
Hudson, S., M.D., On Epidemic Cerebro-Spinal Meningitis .......... 269
Hyde, Jas. N., M.D., Integumentary and Generative Organs .......... 171
  Syphilis, Unity or Duality of .......... 530
  Lanfranc, of Milan. His Brief .......... 705
Hydrate Chloral, On Chronic Poisoning with—Dr. Ludwig Kirn .......... 487
Hygiene and Materia Medica .......... 250

**I**

Illinois State Medical Society...315, 510, 575
Imposition on Life Insurance .......... 637
Infantile Diarrhœa—Ira E. Oatman, M.D. .......... 336
Influenza, Horse .......... 320
Insane Females, Retreat for .......... 61
Integumentary and Generative Organs—Jas. N. Hyde, M.D. .......... 171
Insanity, Premonitory Symptoms of—Horatio R. Bigelow .......... 717
Iodide of Potassium in Erysipelas—A.H. Hiatt, M.D. .......... 593
Iris, Rare Abnormal Condition of—E.L. Holmes, M.D. .......... 330

**J**

Jackson, A. Reeves, M.D., Annual Report on Obstetrics and Diseases of Women .......... 146
  Unsuccessful Attempt to Remove a Fibrous Tumor .......... 159
Johnson, H. A., M.D., Statistics of Vaccination ..........56, 57
  Case of Rheumatic Carditis .......... 500
Joy, Prof. C. A., Death of Baron Liebig 428

**K**

Kearney, T. H., On Nephrotomy ...... 114
King, W. Henry, M.D., Concerning Amalgam Plugs .......... 523
Kinsman, D. N., M.D., Clinical Examination of Children .......... 286
Kirn, Dr. Ludwig, On Chronic Poisoning with Chloral Hydrate .......... 487
Kittell, Geo. W., M.D., Injury from Glass 324
Knox, J. Suydam, Case of Strangulated Hernia .......... 458

**L**

Lanfranc, of Milan. His Brief—Jas. Nevins Hyde, M.D. .......... 705
Liebig, Baron, Death of—Prof. C. A. Joy 428
Life Insurance, Imposition on .......... 637
Life Insurance .......... 639
Liver, Wound of—G. W. Stewart, M.D. .. 520
Locomotor Ataxy, Case of, Benefited by Use of Nitrate of Silver—G. C. Paoli, M.D. .......... 592
Loot—
  A Physician's Diary .......... 447
  Attempted Reduction of Dislocation of Shoulder Joint .......... 317

Loot— PAGE
  Cod Liver Oil and Lacto-Phosphate of Lime .......... 445
  Dengue, or Break-Bone Fever .......... 444
  Elevating the Standard .......... 316
  Normal Ovariotomy .......... 702
  Treatment of Nervous Aphonia and Chronic Pharyngitis .......... 446
  Treatment of Traumatic Neuralgia ... 634
  What is Homœopathy? .......... 318
  Why Insanity Increases .......... 446
Lumbricus, Discharged Through an Abscess .......... 120
Lunatics, Are they Criminals? .......... 700
Lyman, H. M., M.D., Interview with Brown, the "Mind Reader" .......... 565

**M**

Mack, Dr., Nature and Treatment of Vaginismus .......... 399
Mansfelde, Alex.S.von, Cholera Infantum 453
Marsh, W. R., Obituary of .......... 761
Marsh Fever, How Produced .......... 657
McClelland, M. A., M.D., Civil Malpractice ..........65, 129, 193
McFarland, Andrew, M.D., Private Retreat for Insane Females .......... 61
Medicine and Surgery, Bill to Promote 126, 758
Meningitis, Epidemic Cerebro-Spinal 269, 345
Mercury and Iodide of Potassium in Syphilis .......... 635
Microscopical Examinations of Urine ... 342
"Mind Reader"—H. M. Lyman, M.D. .. 565
Minich, A. K., M.D., Paresis .......... 219
Montgomery, E., M.D., Epidemic Cerebro-Spinal Meningitis .......... 345
Murray, W. W., M.D., Gelseminum as an Antiperiodic .......... 355

**N**

Nephrotomy—Thos. H. Kearney, M.D. . 114
Neuralgia—Walter Hay, M.D. ........ 4, 97
Neuralgia of the Testicle .......... 239
Neuralgia, Case of Traumatic, Treated. 542
Neuralgia, Traumatic, Treatment ...... 634
Nichols, J. E., M.D., Removal of External Tumors .......... 203
  Shortening and Bending in Fractures of the Femur .......... 274
Night Diet .......... 119
Nose, Barking of the .......... 668
Nursing of Children .......... 290

**O**

Oatman, Ira E., M.D., Infantile Diarrhœa .......... 336
Obituary, Dr. Wm. Henry Palmer ...... 256
  Wells Ransom Marsh, M.D. .......... 761
O'Brien, J. E., M.D., Puerperal Fever .. 526
  Ante-Partum Prolapsus of the Cord. 656
Ophthalmia, Diphtheritic—F. C. Hotz, M.D. .......... 16
Our Publishers .......... 124
Ozone and Cholera .......... 672

**P**

Paget, Sir James, On Bed Sores .......... 424
Palmer, Wm. H., Obituary of .......... 256
Paoli, G. C., M.D., Fatal Case of Placenta Prævia .......... 105
  Case of Locomotor Ataxy .......... 592
Paralysis, Case of Facial—G. M. Smith, M.D. .......... 560
Paresis—Andrew K. Minich, M.D. ...... 219

PAGE

Paresis, General, Some Conclusions in regard to—H. R. Bigelow.... ...... 403
Paresis, General, Case of—J. B. Stonehouse, Jr., M.D.................... 743
Paroxysmal Fevers.................... 657
Pascale, De, Dr., Use of Dry-Powdered Blood.......................... 435
Pathological Society, London, Discussion on Anatomical Relations of Pulmonary Phthisis to Tubercle—Z. Collins McElroy, M.D.................... 723
Petition, The Right of. ............... 190
Philadelphia Obstetrical Society, Appeal of ............................ 255
Phthisis, as Related to Syphilis and Scrofula—H. C. Hand, M.D........ 411
Physical Education and Training of Children—H. W. Boone, M.D.......... 38
Pillsbury, B., M.D., On Amalgam Plugs 596
Placenta Prævia...................... 105
Pleuritic Exudations—C. Rauschenberg, M.D ............................ 232
Pneumogastric, Notes on—J. F. A. Adams, M.D......................618, 676
Pneumonia............................ 675
Poisoning, Uræmic and Alcoholic—S. Rogers, M.D.................. .... 296
Poisoning from Corrosive Sublimate—J. Payne, D.D.S........ ........... 397
Population, Law of—Nathan Allen..... 332
Potassium in Syphilis................. 635
Private Retreat for Insane Females..... 61
Publishers' Notice.................... 757
Puerperal Fever—J. E. O'Brien, M.D.. 526
Putnam, Jas. J., Method of Examining the Brain.......................... 682

R

Rauschenberg, C., M.D., Pleuritic Exudations................................ 232
Reports of Medical Societies—
American Medical Association....251, 364
American Public Health Association.. 704
Brainard Medical Society............ 640
Cass County (Indiana) Medical Society 640
DeWitt County Medical Society...... 448
The Illinois State Medical Society 315, 510, 575
The Chicago Society of Physicians and Surgeons.............146, 385, 590, 742
The Central Illinois Medical Society.. 127
Woodford County Medical Association 760
Reynolds, Dr. J. W. B., Dropsy of the Amnios .......................... 54
Rheumatic Carditis, A Case of—H. A. Johnson, M.D.................... 500
Rheumatism—Jas. E. Whitmire, M.D... 207
Rheumatism, On Acute Articular—Austin Flint, M.D.................... 357
Ricord on Syphilis.... ............... 46
Rogers, S., M.D., On Uræmic and Alcoholic Poisoning.................... 296
Rush Medical College Concours........ 63
Rush Medical College, Thirtieth Annual Commencement of.............. 191

S

Sanitary ............................ 570
Sansom, A. Ernest, M.D., Disinfection of Air ............................ 279
Schirrhous Affections of the Stomach—X. T. Bates, M.D.................. 398
Seguin, E. C., M.D., Case of Traumatic Neuralgia treated.................. 542
Septicæmia—J. W. Freer, M.D......... 106
Sequard, C. E. Brown, Diseases of the Brain ............................ 468
Shoulder Joint, Attempted Reduction of Dislocation of .................... 317
Skill ................................. 671
Skin Grafting ........................ 63
Smith, G. M., M.D., Case of Facial Paralysis ........................... 560
Specific Diseases, Spread of............ 657
Speculum, The use of ................ 257
Statistics of England, Sanitary—T. D. Crothers ............................ 433
Stewart, G. W., M.D., Case of Wound of the Liver........................ 520
Stomach, Case of Chronic Inflammation of—Drs. A. Fisher, James N. Hyde, and I. N. Danforth................. 585
Syphilis, Ricord on ................. 46
Syphilis and Scrofula, Phthisis as related to—H. C. Hand, M.D............ 411
Syphilis, Unity or Duality of—J. Nevins Hyde, M.D.................. 530
Syphilis, Mercury and Iodide of Potassium in ........................... 635

T

Tactus Eruditus....................... 673
Testicle, Neuralgia of................. 239
Toner Lectures ....................... 312
Translations from Foreign Journals—J. H. Etheridge, M.D................ 668
Traumatic Neuralgia, Case of........... 542
Treatment of......................... 654
Traumatic Tetanus, Successfully Treated with Calabar Bean—J. A. Duncan, M.D.............................. 686
Tumors, Removal of External—J. E. Nichols, M.D...................... 203
Tyson, James, M.D., Microscopical Examinations of Urine................ 342

U

Umbilical Hemorrhage, Death from—Norman Bridge, M.D.............. 595
Unhealthy Business................... 541
Urine, Microscopical Examinations of—Jas. Tyson, M.D.................. 342
Uterus, Varicose Hemorrhage from...... 406

V

Vaccine Virus ........................ 310
Vaccinations, Successful—H.A. Johnson, M.D ............................. 56
Vaginismus, Nature and Treatment of—Dr. Mack ...................... 399
Vance, R. A., M.D., Ophthalmoscopic Appearances in Cases of Exophthalmic Goitre ........................ 449
Varicose Hemorrhage from the Cervical Zone of the Uterus—G. C. P. Murray, M.D........................... 406
Variola, Congenital, in the Fœtus—Philip Adolphus, M.D..................... 278
Ventilation and Warming .............. 363

W

Wey, W. C., M.D., The Certificates of Family Physicians.................. 562
Whitmire, J. S., M.D., Rheumatism.... 207
Whooping Cough and Quinine—B. F. Dawson, M.D........................ 224

Y

Yates Co. Crystal Springs .............. 125

RENEW YOUR SUBSCRIPTIONS.

**PARTICULAR NOTICE.**

***THE JOURNAL will not be sent, after the present number, to any who have not renewed their subscriptions.***

THREE DOLLARS A YEAR. SPECIMEN COPIES, 30 CENTS.

VOL. XXX. January, 1873. NUMBER I.

THE

### Chicago Medical Journal.

**Remit $3.00 for the new volume, commencing January, 1873.**

**Complete Sets for last year can be furnished for $3.00.**

